# Bench to Bed Evidences for Pharmacokinetic and Pharmacodynamic Interactions Involving Oseltamivir and Chinese Medicine

**DOI:** 10.1155/2014/354172

**Published:** 2014-01-09

**Authors:** Qi Chang, Siukwan Wo, Karry L. K. Ngai, Xiaoan Wang, Benny Fok, Teresa M. Ngan, Vivian T. Wong, Thomas Y. K. Chan, Vincent H. L. Lee, Brian Tomlinson, Paul K. S. Chan, Moses S. S. Chow, Zhong Zuo

**Affiliations:** ^1^Institute of Medicinal Plant Development, Chinese Academy of Medical Science and Peking Union Medical College, Beijing 100193, China; ^2^School of Pharmacy, Faculty of Medicine, The Chinese University of Hong Kong, Shatin, New Territories, Hong Kong; ^3^Department of Microbiology, Faculty of Medicine, The Chinese University of Hong Kong, Shatin, New Territories, Hong Kong; ^4^Department of Medicine and Therapeutics, Faculty of Medicine, The Chinese University of Hong Kong, Shatin, New Territories, Hong Kong; ^5^Hospital Authority, Hong Kong; ^6^Center for Advancement of Drug Research and Evaluation, College of Pharmacy, Western University of Health Sciences, Pomona, CA 91766-1854, USA

## Abstract

Oseltamivir (OA), an ethyl ester prodrug of oseltamivir carboxylate (OC), is clinically used as a potent and selective inhibitor of neuraminidase. Chinese medicines have been advocated to combine with conventional drug for avian influenza. The current study aims to investigate the potential pharmacokinetic and pharmacodynamic interactions of a Chinese medicine formula, namely, Yin Qiao San and Sang Ju Yin (CMF1), commonly used for anti-influenza in combination with OA in both rat and human, and to reveal the underlined mechanisms. It was found that although *C*_max_, AUC and urinary recovery of OC, as well as metabolic ratio (AUC_OC_/AUC_OA_), were significantly decreased in a dose-dependent manner following combination use of CMF1 and OA in rat studies (*P* < 0.01), such coadministration in 14 healthy volunteers only resulted in a trend of minor decrease in the related parameters. Further mechanistic studies found that although CMF1 could reduce absorption and metabolism of OA, it appears to enhance viral inhibition of OA (*P* < 0.01). In summary, although there was potential interaction between OA and CMF1 found in rat studies, its clinical impact was expected to be minimal. The coadministration of OA and CMF1 at the clinical recommended dosages is, therefore, considered to be safe.

## 1. Introduction

Oseltamivir (OA) is clinically used as a potent and selective inhibitor of neuraminidase essential for replication of influenza A and B viruses. The normal adult dose of OA for the treatment of avian influenza is 75 mg orally twice a day for 5 days. Following 50 mg doses, the maximum plasma oseltamivir carboxylate concentration is about 230 *μ*g/L, which is above of 50% inhibitory concentrations (IC_50_) of many influenza A viruses [1]. The pharmacokinetics of both OA and its active metabolite oseltamivir carboxylate (OC) have been studied in young healthy adults and children, as well as elderly subjects [1–4]. Following oral administration, OA is rapidly absorbed and extensively converted to OC, primarily by hepatic carboxylesterase enzymes, resulting in a much higher concentration *in vivo* than OA. The absolute oral bioavailability of OC from orally administered OA is 80% with a half-life of 6–10 hours and food has no significant effect on its bioavailability [1, 5].

The potential advantage of OA in combination with Chinese medicine (CM) is of interest since avian influenza can be deadly and it is an important health care goal in many Asian countries. In Hong Kong, the Hospital Authority Central Committee on Infectious Disease and Infection Control Branch Centre for Health Protection has jointly recommended the drug OA (Tamiflu) for prophylaxis and treatment of avian influenza. In addition, a panel of the CM experts from HA together with the Task Force on Herb-Drug Interaction Research has recommended four specific CM formulae for the prophylaxis and treatment of influenza with OA. It is expected that many Hong Kong citizens will be prescribed with such “western” medicine (WM) and CM for avian influenza if there is an outbreak. Whether coadministration of the CM formulae as recommended by HA expert will cause any change in plasma oseltamivir carboxylate concentration or whether there is an additive antiviral effect with the combination is unknown. Although there is a report on the effect of a few CM (*Flos Lonicerae, Folium Perillae, Radix isatidis*) on OA [6], the findings are mainly from *in vitro* cell studies. The present study aims to determine, in animal and human studies, the potential pharmacokinetic and pharmacodynamic interactions of OA in combination with the most recognized and thirteen herb containing CM formulae (CMF1, Table 1), which is a combined formula of two traditional Chinese herb preparations, Yin Qiao San and Sang Ju Yin, for avian influenza as recommended by a CM expert panel from the HA in Hong Kong.

## 2. Materials and Methods

### 2.1. Chemicals and Reagents

OA (RO-64-0796) and D-tartrate salt of OC (RO-64-0802) were kindly provided by F. Hoffmann-La Roche Ltd. (Switzerland). Cephalexin hydrate was purchased from Sigma (USP Science, Rockville, MD, USA). Dichlorvos (as carboxylase inhibitor) with purity of 99.4% was purchased from Riedel-de Haën (Germany). Methanol (HPLC grade) was purchased from Merck KGaA (Germany). Unless specified elsewhere, all reagents were used without further purification. Distilled and deionized water (ddH2O) was prepared from Millipore water purification system (Millipore, Milford, USA). Tamiflu 75 mg capsule (batch number B1280B01) (Hong Kong registration number HK-46484) was manufactured by F. Hoffmann-La Roche Ltd.

For cell culture, Dulbecco's modified Eagle's medium, fetal bovine serum, 0.05% Trypsin-EDTA, penicillin-streptomycin, and nonessential amino acids were obtained from Gibco BRL (Carlsbad, CA, USA) and Life Technologies (Grand Island, NY, USA). Phosphate buffered saline tablets were purchased from Sigma.

CMF1 was manufactured by Purapharm (Nanning) Pharmaceuticals Co. Ltd. in accordance with GMP standard. CMF1 (batch number A090943-01) was formulated as granules and received in package of 10 g granules per sachet for human study. The safety measures including heavy metals (arsenic, lead, mercury, and cadmium), microbial examination, and pesticides residue of the CM products were conducted by an independent Hong Kong accredited laboratory and were found to comply with the 2005 Hong Kong Traditional Chinese Medicine requirements. HPLC/DAD was used to obtain a chemical profile of potential active components of CMF1. Briefly, 30 mg of CMF1 powder was accurately weighted into a glass container with tight cap, and 5 mL of methanol water (50 : 50 v/v) was added and sonicated for 15 min for extraction. After centrifugation at 13,000 rpm for 10 min, the supernatant (10 *μ*L) was then injected into HPLC-DAD (Waters, Milford, MA, USA) for assay of active components. The sample was separated by a Thermo ODS Hypersil column (4.6 × 250 mm, 5 *μ*m) connected to a ODS guard column (Thermo). The mobile phase consisted of 0.2% formic acid in water (solvent A) and in acetonitrile (solvent B) with linear gradient elution at a flow rate of 1 mL/min. Solvent B was set at 5% from 0 to 5 min and increased to 40% from 5 to 45 min and then back to 5% in 10 min with 5 min. The PDA detector was set for collection of spectral data from 210 nm to 400 nm. The contents of arctiin and forsythoside A, the identified marker components for CMF1, are 3.54 mg/g and 0.364 mg/g, respectively, which fulfilled the requirement of related formula in the Chinese Pharmacopoeia [7].

### 2.2. Drug Administration and Samplings in Rats

The study was approved by the Animal Ethics Committee of The Chinese University of Hong Kong. Male Sprague-Dawley rats (230–250 g) were utilized and supplied by the Laboratory Animal Service Center at The Chinese University of Hong Kong. The rats were housed under standard conditions of temperature, humidity, and light and randomly divided into six groups with 10–12 rats in each group. In order to achieve full pharmacokinetics profiles of both OA and OC, our preliminary experiments suggested a dose of 30 mg/kg for OA oral administrations. The dose of 1.95 g/kg CMF1 is calculated based on the human dose recommended by the Chinese physicians and a doubled dose of CMF1 at 3.90 mg/kg is also used in the current study due to the potential large dosing range for Chinese medicine adopted in the practice. The rats in Group 1 received OA alone (30 mg/kg), and those in Groups 2 and 3 received OA (30 mg/kg) in combination with CMF1 at low (1.95 g/kg) and high (3.90 g/kg) doses, respectively. Rats in Groups 4 and 5 were only treated with CMF1 at low (1.95 g/kg) and high (3.90 g/kg) doses, respectively, whereas Group 6 rats only received regular diet without OA or CMF1.

For dosing, OA (4 mg/mL) was freshly prepared by dissolving it in water and orally given to rats by gavage, bid (twice daily) for 4 days (9:00am and 6:00pm for each day), and CMF1 was also freshly suspended in water and then orally given to rats 2 h later of OA dosing (11:00 am and 8:00 pm for each day).

For Groups of 1 to 3, a surgery for jugular vein cannulation was performed 1 h after CMF1 second dosing on day 4. A polyethylene catheter (0.50 mm ID, 1.00 mm OD, Portex Limited, Hythe, Kent, England) was cannulated into the right jugular vein under anesthesia. After surgery, the rat was placed in separated metabolic cage and allowed to recover and fasted overnight with free access to water. In the morning of day 5, blood samples (0.2 mL each) of the rats in the Groups 1–3 were collected via the catheter at 0, 15, 30, 60, 90, 120, 180, 240, 360, and 480 min after dosing of OA. After which, 0.2 mL of normal saline containing 20 units/mL of heparin was then injected into the catheter to flush the catheter. The collected blood samples were immediately placed in heparinized tubes containing dichlorvos (5 *μ*L of 8 mg/mL dichlorvos in normal saline) for inhibition of carboxylesterase [8], followed by centrifugation to obtain the plasma and stored at −80°C. Urine samples were collected over 8 h postdose and combined with water used for rinsing the metabolic cage, further diluted to 200 mL, and then stored at −80°C until assay.

Following the last blood sampling, the rats in Groups 1–3 received the last dose of OA or OA together with CMF1 similar to above dose, respectively. At 90 min after dosing (i.e., absorption had taken place), all rats were sacrificed and ~2 mL plasma was collected for determination of antiviral activity. Rats in Groups 4 to 5 were sacrificed at 90 min after last dosing and ~2 mL of plasma was collected for determination of antiviral activity. The plasma samples collected from the rats in Group 6 (without any treatment) were served as negative control.

### 2.3. Human Study

#### 2.3.1. Ethics

Prior to the clinical study, the ethics approval was obtained from the Joint Chinese University of Hong Kong-New Territories East Cluster Clinical Research Ethics Committee. The clinical study was conducted according to Good Clinical Practice (GCP) and ICH guidelines and the Declaration of Helsinki. All subjects were fully informed about the study and a written informed consent was obtained from each subject prior to the study.

#### 2.3.2. Subjects

Normal healthy Chinese male subjects aged 20–45 years were recruited in this study. Subjects were excluded if they had a history of clinically significant hepatic, renal, biliary, cardiovascular, gastrointestinal, haematologic and other chronic and acute diseases within 3 months prior to the study; had clinically relevant abnormality in physical examination, ECG evaluation, urine test, blood chemistry or haematological test during screening test; received any prescription or hypersensitivity to Tamiflu or related CM formulae/herbal components; a history of smoking, drug or abuse of alcohol; blood donation within 4 weeks prior to the start of study.

The screening process included physical examination, ECG evaluation, urinalysis, blood chemistry, and haematological tests. During the study, subjects were abstained from any prescription or nonprescription medications 2 weeks before and throughout the study; alcohol, grapefruit juice, caffeine, or xanthine-containing foods or beverages for 72 h prior to and during sampling; smoking for 72 h prior to and during sampling.

#### 2.3.3. Clinical Study Design

A sample size of 14 was calculated by assuming that a 15% difference (based on our preliminary study) in the mean pharmacokinetic parameter is significant between 2 groups (WM *versus* WM + CMF) and a 30% standard deviation to achieve 80% power at *α* = 0.05. The study was conducted using a single-center, randomized, open-labeled, multiple dose (5 days), two-treatment, two-period, two-sequence crossover design. Subjects were randomized to one of two groups (Groups 1A and 1B) and received either western medicine (WM) alone or in combination with CMF1. Group 1A received WM first followed by WM in combination of CMF1, while Group 1B received WM in combination of CMF1 first followed by WM. Each subject underwent two treatment sessions (periods I and II), and each session consists of 5-day treatment (twice daily for day 1 to day 4, morning dose for day 5). The two treatment sessions were separated by a washout period of 2 weeks. 250 mL water was used for WM (Tamiflu capsule, 75 mg per dose) administration. CMF1 (10 g extracts per dose) was mixed to 250 mL water before administration (2 h after receiving WM, based on the common practice for combination use of western drugs and Chinese medicines recommended by local practitioners). Subjects were fast for 10 h before and 4 h after drug administration on day 5 of each treatment session. Drinking water was not allowed from 1 h predose to 1 h postdose except that needed for drug dosing. Meals were standardized and consumed at 4 h and 10 h postdosing.

#### 2.3.4. Blood and Urine Collection

All blood and urine collection was taken on day 5 of each treatment session. Venous blood samples were collected at pre-dose (0 h) and at 0.5, 1, 2, 3, 4, 5, 6, 8, 10, and 12 h post-dose. Blood samples were collected from a catheter, which was placed in the forearm vein before dosing. At the specified time, 5 mL of blood was drawn (except for 0 time and at 2 h which was 10 mL each for additional antiviral activity determination) and stored in vacuette lithium heparin tubes (Greiner Bio-One). Dichlorvos (a carboxylase inhibitor) was then added (200 *μ*g/mL) into the blood samples (except for those used for antiviral activity determination) to prevent *in vitro* hydrolysis from OA to OC [8–10]. Plasma samples were collected after centrifugation and then stored at −80°C until assay. Urine was also collected (and the volume was recorded) at pre-dose, 0–4, 4–8, and 8–12 h intervals. The urine samples (~10 mL) were stored at −80°C until assay.

### 2.4. Determination of OA and OC in Plasma and Urine by LC/MS/MS

The rat/human plasma and urine samples were treated and analyzed by an LC/MS/MS system as previously described [10]. Briefly, 200 *μ*L plasma/urine samples was mixed with cephalexin hydrate (internal standard, IS) working solution (final 2 *μ*g/mL) and acidified with 1 mL of 10% perchloric acid in water (if necessary, dilution with blank human urine was required for urine samples). After mixing and centrifugation, the supernatant was loaded in prewashed Oasis MCX cartridge (1 cc, 30 mg, Waters) and the cartridge was rinsed subsequently with 1% formic acid, water, and methanol followed by vacuum dried for 20 min. The analytes were then eluted with 1 mL of 1% ammonia in methanol. The eluting solvent was dried by a vacuum concentrator and the residue was reconstituted with 200 *μ*L of 0.1% formic acid : methanol (1 : 1 v/v) prior to HPLC/MS/MS analysis using an ABI 2000 Q-Trap triple quadrupole mass spectrometer (Applied Biosystems) coupled with PE-200 series micropumps and autosampler (Perkin-Elmer). The chromatographic separation was achieved by using a Nova-Pak CN HP column (75 × 3.9 mm i.d., 4 *μ*m particle size, Waters) and the HPLC solvent system consisted of methanol (A) and 0.1% formic acid in water (B), with 50% A (for plasma samples) or 60% A (for urine samples) at 1 mL/min. The temperatures of autosampler and the analytical column were set at 4°C and ambient, respectively, and the sample injection volume was 20 *μ*L. Prior to the mass spectrometric system, 60% of the LC eluent was split off and only 40% of the eluent was introduced into the ESI source.

The mass spectrometer was operated at positive ionization mode. Ion spray voltage was set to 5500 V; heater probe temperature was set at 400°C; nitrogen was used as nebulizer (30 psi), heater (70 psi), curtain (30 psi), and collision gas (medium). Other instrumental parameters were analyte specific and were optimized prior to analysis. Data acquisition was conducted at multiple reaction monitoring (MRM) mode, with *m/z* 313 → *m/z* 166 for O,* m/z* 285 → *m/z* 138 for OC, and *m/z* 348 → *m/z* 158 for IS. Dwell time was set at 300 ms for each channel.

Calibration standards were prepared by spiking 200 *μ*L blank human plasma (premixed with dichlorvos to a final concentration of 200 *μ*g/mL)/200 *μ*L blank human urine with 20 *μ*L each of working standard mixture and internal standard solution. The linearity of analytes (as OA phosphate or OC tartrate) was 2–1000 ng/mL (OA) and 10–10000 ng/mL (OC) in plasma and 6–1000 ng/mL (OA) and 30–10000 ng/mL (OC) in urine. The LOQ of OA and OC, defined as the signal-to-noise ratio ≥5 and being reproducible with precision of 20% RSD and accuracy between 80% and 120%, was the lowest concentration of the calibration curve.

The method validation was conducted with reference to the Guidance for Industry, Bioanalytical Method Validation from USFDA (May 2001) with satisfied accuracy and precision of OA and OC at low, medium, and high concentration levels in either plasma (6, 80, and 750 ng/mL for OA, 20, 800, and 7500 ng/mL for OC) or urine (15, 150, and 750 ng/mL for OA, 60, 1500, and 7500 ng/mL for OC) were found to comply with the criteria of accuracy (within 15% bias) and precision (within 15% RSD) as stated in the guidance. The extraction recoveries of OA (87%–109%), OC (73%–81%), and IS (70%–88%) in both plasma and urine were consistent over the concentration range studied. The analytes in both plasma and urine under three freeze (−80°C) thaw (room temperature) cycles, short term (2 h at ambient), and in autosampler (at 4°C for 12 h for plasma and 6 h for urine samples) were found to be stable (accuracy of 88.0%–106.1%) and reproducible (within 10.1% RSD) over the concentration range investigated.

### 2.5. Antiviral Effects Measurement

#### 2.5.1. Cells and Viruses

Madin-Darby canine kidney (MDCK) cells were cultured in Eagle's minimum essential medium (MEM) (Invitrogen, California, USA). Media were supplemented with 10% fetal bovine serum (FBS) (Invitrogen, California, USA), except the assays of influenza virus. Infections with human influenza A H3N2 virus (A/HongKong/CUHK-22910/2004) were carried out in serum-free medium formulated with 1 *μ*g/mL of trypsin treated with tolylsulfonyl phenylalanyl chloromethyl ketone (TPCK-treated trypsin) (Sigma-Aldrich, Munich, Germany).

#### 2.5.2. Plaque Reduction Assay

The antiviral effects of rat plasma samples at 120 min after oral administration of OA or OA in combination with CMF1 were evaluated by the plaque reduction assay [11]. Briefly, the plasma samples obtained from Groups 1 to 6 were first ultrafiltrated at 4°C using an Amicon Ultra 3 K filter unit (Millipore) to remove protein and then diluted with serum-free medium in 250-fold dilution. An equal volume (0.25 mL) of diluted plasma was mixed with virus culture medium containing 400 PFU/mL and incubated for 1 h at 37°C. Confluent monolayer of MDCK cells in 24-well plate (Nunc, Denmark) was washed with infectious medium and inoculated with 0.5 mL plasma-containing virus mixture. After 1 h of viral absorption at 37°C, virus inoculums were removed before adding 0.5 mL agarose overlay medium containing 0.4% agarose and 500-fold diluted plasma. Duplicates of each plasma sample, virus control, and cell control were performed in each experiment. Plaques were stained with neutral red staining (0.05%) after 24 h incubation at 37°C. The plaques were counted and anti-viral activity was calculated as the percentage of virus control. Blank rat plasma spiked with 2 *μ*g/mL of OC was treated as mentioned above and served as the positive control for the assay.

Similarly, human plasma samples (~2 mL) collected on day 5 at pre-dose (0 h) and 2 h after medications in human were ultrafiltered before conducting the plaque reduction assay. Prior to analysis, the human plasma filtrate was diluted in 1 : 10 with maintenance medium and the diluted samples were mixed with equal volume of virus. Influenza virus H3N2 strain was used for the assay. The results were presented as percentage inhibition (*versus* control, i.e., drug-free plasma filtrate).

### 2.6. Mechanistic Studies on the Effect of CMF1 on the Metabolism and Absorption of OA

#### 2.6.1. Effect of CMF1 on the Hydrolysis of OA in Rat Plasma

For testing the metabolic activity of OA in rat plasma, 20 male Sprague-Dawley rats (230–250 g) were sacrificed with an intramuscular injection of a mixture of ketamine (60 mg/kg) and xylazine (6 mg/kg). Rat blood was obtained via cardiac puncture with a 10 mL syringe containing 0.1 mL heparin (5000 I.U./mL) followed by centrifugation at 13000 rpm for 5 min. The obtained plasma from all rats was pooled together and stored at −80°C for enzyme incubation experiments.

Rat plasma with a volume of 400 *μ*L was spiked with 4 *μ*L CMF1, which was dissolved in DMSO (control group was spiked with DMSO only). Final tested concentrations for CMF1 ranged from 25 *μ*g/mL to 300 *μ*g/mL. Working solutions of OA, in H_2_O, were then spiked into the above reaction mixture to reach a final concentration of 5 *μ*g/mL (for reactions at room temperature) or 10 *μ*g/mL (for reactions at 37°C). The final mixture was incubated for 60 min (for reactions at room temperature) or 30 min (for reactions at 37°C) and terminated by adding 10 *μ*L dichlorvos (2 mg/mL) into 100 *μ*L reaction mixture. Samples are prepared and analyzed by a developed method with modifications [10].

#### 2.6.2. Effect of CMF on the Absorption of OA in Rat *In Situ* Intestinal Perfusion Model

In view of the consistent effect of CMF1 on OA and OC, further confirmation was performed using rat *in situ* intestinal perfusion model as described previously [12]. Perfusion concentrations were set at 6.8 mg/mL for CMF1. The flow rate of perfusate applied to the intestinal lumen was set at 0.3 mL/min. Samples obtained from the mesenteric vein were collected into the preweighted 2 mL centrifuge tubes (each containing 30 *μ*L of 10 mg/mL saline solution of dichlorvos) at every 5 min. All collected samples were weighted and centrifuged at 13,000 rpm for 4 min immediately. The plasma samples were stored at −80°C refrigerator until further treated by SPE and analyzed by LC/MS/MS assay as described above.

### 2.7. Data Analyses

#### 2.7.1. Pharmacokinetics and Enzyme Kinetics Parameters

The plasma/urine OA and OC concentrations versus time profiles were analyzed using WinNonlin software standard edition version 2.1 (Pharsight Corporation). The noncompartmental model was employed to estimate the pharmacokinetic parameters including time of maximum observed concentration (*T*_max_), concentration corresponding to *T*_max_ (*C*_max_), terminal half-life (*t*_1/2_) and area under curve from time zero to the last sampling time (AUC_0–*t*_) for plasma samples, and the 12 h cumulative amount of analytes (A_e_) excreted in urine. In human study, trough concentration (*C*_trough_) was defined as the minimum concentration obtained and was obtained at 12 h post-dose. The renal clearance (Cl_r_) was calculated as the 12 h cumulative amount of analyte in urine divided by the plasma AUC_0–12 h_. The AUC ratio of OC/OA in plasma and the 12 h cumulative amount ratio of OC/OA in urine were also evaluated.

Percentage of inhibition of CMF1 on the metabolism of OA is calculated according to the following equation: % inhibition = [1 − (OC/OA)_CMF  treatment_/(OC/OA)_control_] × 100, in which OC/OA refers to the ratio of OC and OA concentration in the incubated samples.

Percentage of inhibition for antivirus activity in the plaque reduction assay was calculated by the following equation: % inhibition contributed by treatment = (*N*_virus  control_ − *N*_treatment_)/*N*_virus  control  _ × 100, where *N* refers the number of plaques.

#### 2.7.2. Statistic Analyses

For rat studies, all data obtained were expressed as mean ± standard deviation. Unpaired Students *t*-test was used to compare the pharmacokinetic parameters obtained between the two different treatment groups. ANOVA followed by post hoc test was used for the antiviral effect comparisons among different treatment groups. A *P* < 0.05 was considered to be significant.

In human studies, the comparison of WM + CMF1 to WM alone treatments was evaluated using a 90% confidence intervals (90% CI) approach (USFDA guideline, Drug Interactions Studies, September 2006) [13]. Analysis of variance (ANOVA) using General Linear Model (GLM) procedure was performed on logarithmically (natural logarithm) transformed *C*_max_, AUC_0–12 h_ and the 12 h cumulative amount of OA and OC, as well as the AUC_0–12 h_ ratio and 12 h cumulative amount ratio of OC/OA. The statistical model included terms describing the effects attributable to sequence, subject (nested in sequence), period, and treatment (formulation). The 90% confidence intervals (CIs) for the differences in the means of logarithmically transformed *C*_max_, AUC_0–12 h_ and the 12 h cumulative amount of OA and OC, and their AUC_0–12 h_ and 12 h cumulative amount ratio between the combined treatment (WM + CMF1) and western medicine (WM alone) were calculated using two one-sided *t*-tests. The antilogs of the CIs obtained constitute the 90% confidence interval for the geometric mean ratio, that is, (WM + CMF1)/WM, between both treatments. Difference in the median *T*_max_ between both treatments was evaluated using Wilcoxon signed-rank test. For comparison of the human antiviral activities, one-way analysis of variance in conjunction with post hoc Turkey's range test was performed. A *P* value of <0.05 was considered significant.

## 3. Results

### 3.1. Effect of CMF1 on the Pharmacokinetics of OA and OC

#### 3.1.1. Findings from Rat Studies

Effect of CMF1 on the pharmacokinetics of OA and OC in rats was evaluated by comparing the pharmacokinetics profiles of OA and OC among Group 1 to Group 3 that have received various types of treatment with OA. The calculated pharmacokinetic parameters of different treatment groups are shown in Table 2.

In comparing Groups 2 or 3 (two different doses of the CMF1 in combination with OA) with Group 1 (OA alone) (Table 3), the mean plasma concentrations of OA and OC were lower in Groups 2 and 3 than that in Group 1. In Group 3, the peak plasma concentration, AUC, and urinary recovery of OC as well as the AUC ratio of OC *versus* OA were significantly decreased in comparison to those in Group 1 (*P* < 0.01). These results suggested that CMF1 probably inhibited the hydrolysis of OA to OC, and such inhibition effect might be dose-dependent since there was a trend of decreased value in Group 2 compared to Group 1 even though no significant differences were found. Since the urinary excretion of OC was significantly decreased with no change in half-life of OA, a decrease of absorption of OA after administration of high dose of CMF1 cannot be ruled out.

**Figure 1:**
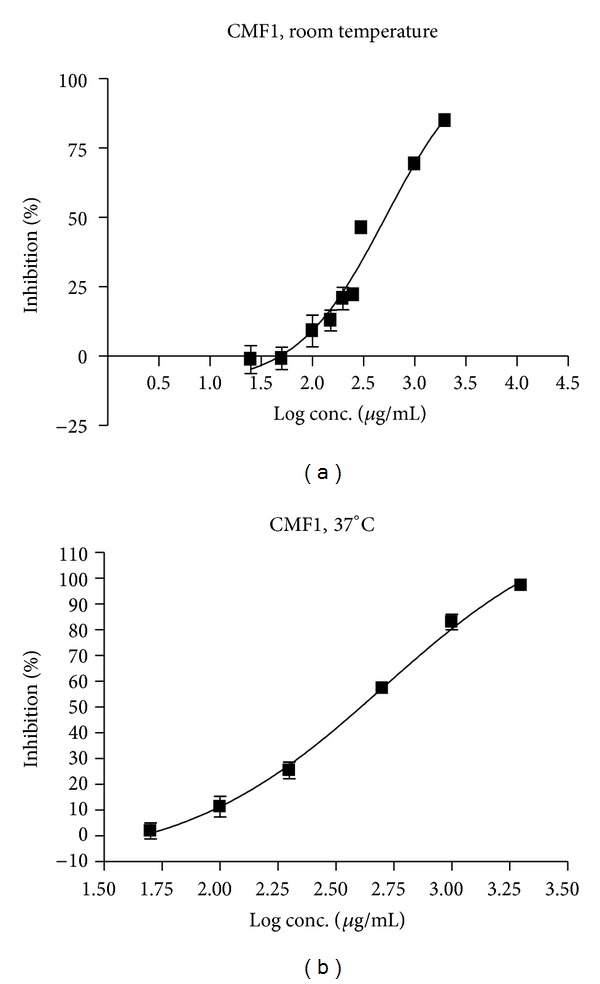
*In vitro* inhibition effect of CMF1 on the hydrolysis metabolism of OA in rat plasma at room temperature (a) and 37°C (b).

**Figure 2:**
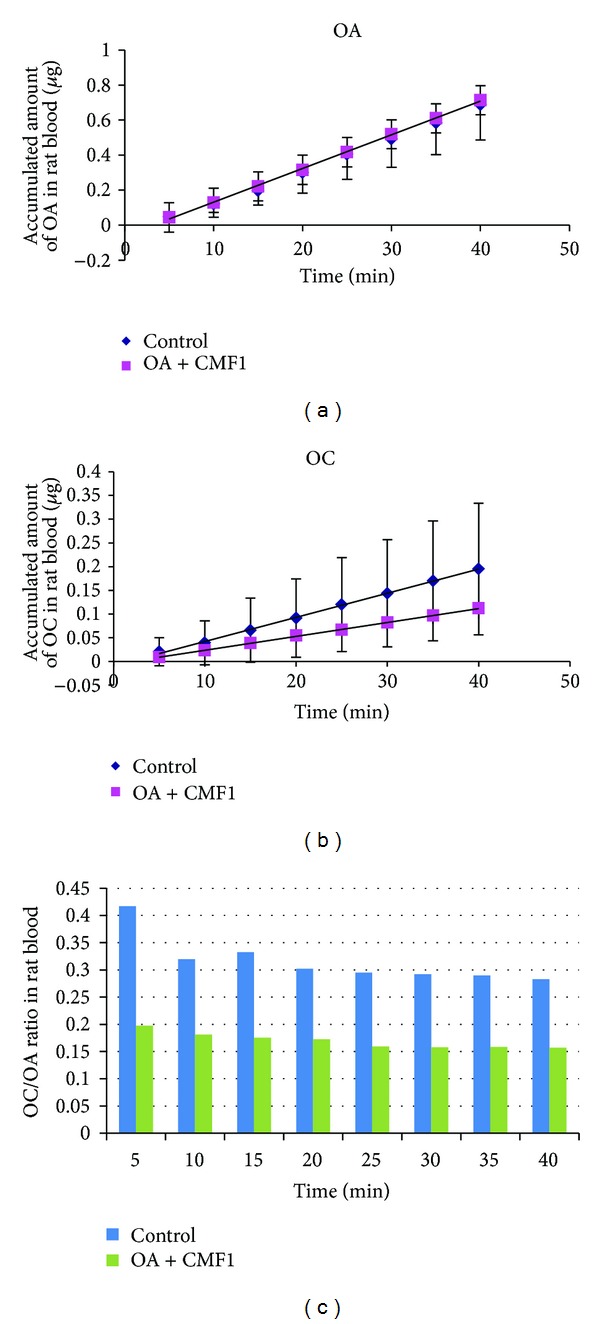
Effect of CMF1 on the accumulated amount of OA (a), OC (b), and OC/OA ratio (c) in rat mesenteric blood samples collected at different time points. Control: intestinal perfusion with OA alone.

Further mechanistic studies revealed that CMF1 exhibited dose-dependent inhibition on the metabolism of OA at both 37°C and room temperature (Figure 1), with a greater % inhibition that occurred at 37°C. Further rat *in situ* intestinal perfusion study indicated that the accumulated OA detected in rat mesenteric blood was not affected by CMF1, whereas accumulation of OC was decreased without statistical significance (Figure 2). However, OC/OA ratio was consistently decreased in presence of CMF1 with time, indicating potential decrease of hydrolysis of OA in presence of CMF1 (Figure 2).

#### 3.1.2. Findings from Human Study

Totally 14 healthy Chinese male adults with average age of 25.2 ± 6.7 years, height of 1.73 ± 0.07 m, weight of 66.9 ± 7.2 kg, and BMI of 22.3 ± 1.8 were recruited and completed in this study, among which 7 subjects were randomly assigned to Group 1A and the other 7 subjects were assigned to Group 1B. Two out of 14 subjects reported mild discomforts during the washout period. These adverse events are mild and unlikely to be related to the study WM and/or WM + CMF1 treatment.

**Figure 3:**
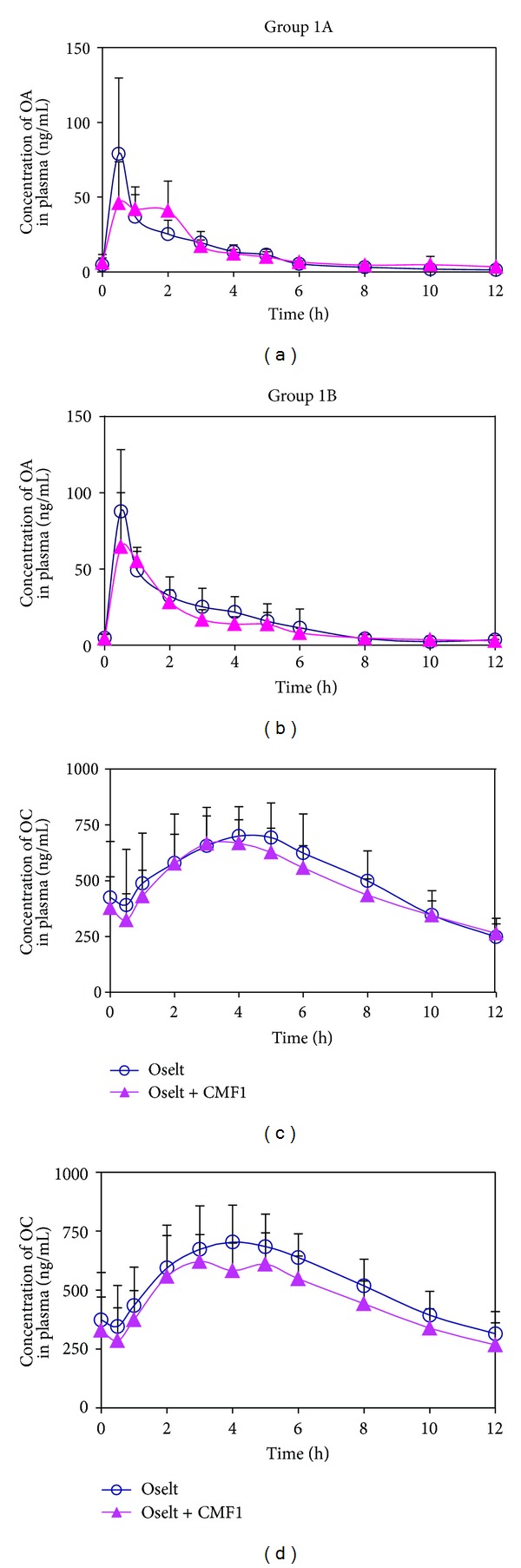
Plasma concentration versus time profiles of OA and OC in Groups 1A (*n* = 7) and 1B (*n* = 7) after oral administrations of oseltamivir (Oselt) alone and oseltamivir in combination with CMF1 (Oselt + CMF1) in 14 Chinese male healthy volunteers.

The plasma concentrations of OA and OC *versus* time profiles are presented in Figure 3 and the related pharmacokinetic parameters of OA and OC in plasma and urine from different treatment groups are presented in Table 3. Upon oral administration of WM, OA (prodrug) was rapidly absorbed and converted to OC (active metabolite). In general, the concentration of OC in plasma was in order of magnitude (~10-fold) higher than the respective OA, indicating that OA was extensively metabolized after drug administration. OA (in plasma) reached peak maximum in ~0.5–1 h, which was considerably faster than that of OC (~4–5 h). The concentration of OA in plasma at pre-dose (0 h) on day 5 of the treatment session and 12 h post-dose (trough concentration) was much smaller than that of OC (Table 3), indicating a faster clearance of OA than OC, and is evidenced by the larger renal clearance of OA (~22 L/h) when compared with OC (~16 L/h). Both OA and OC are in large quantity in urine than in plasma, indicating that both compounds are readily eliminated via renal excretion.

As shown in Table 3 and Figure 3, coadministration of WM + CMF1 generally lowered the mean *C*_max_ of OA and OC in plasma, while only slightly reduction in AUC_0–12 h_ was observed. The cumulative amount of OA excreted in urine remained unchanged in both treatments, though a slightly lower mean value of OC in urine was found when subjects administered with WM + CMF1. There was no significant difference in *T*_max_ between both treatments. The *C*_trough_, *T*_1/2_, and renal clearance of OA and OC were also comparable between both treatments. The geometric mean, geometric mean ratio of (WM + CMF1)/WM, and the 90% confidence intervals of *C*_max_ and AUC_0–12 h_ of OA and OC, and the OC/OA AUC_0–12 h_ are summarized in Table 4. The *C*_max_ (OA) for WM + CMF1 treatment was 21.81% lower than that from WM alone, and the 90% CI ranged from 60.82% to 100.51%, which was lower than the 90% CI criteria from 80% to 125%. More importantly, the geometric mean ratio of AUC_0–12 h_ of OA, the *C*_max_ and AUC_0–12 h_ of the active metabolite (OC), and the OC/OA AUC_0–12 h_ ratio between WM + CMF1 and WM treatments were near unity, and the 90% CI was found to be within the 80%–125% criteria. The point estimates for 12 h cumulative amount (in urine) of OA, OC, and their OC/O ratio were within 85.97% to 99.32% (Table 4). The 90% CI interval for OA excreted (85.56–115.3%) was within the 80%–125% criteria [13], though a slightly lower interval of 90% CI was observed for OC and OC/OA ratio.

### 3.2. Effect of Coadministration of CMF1 with OA on the Antivirus Effect of Tamiflu

#### 3.2.1. Findings from Rat Studies

**Figure 4:**
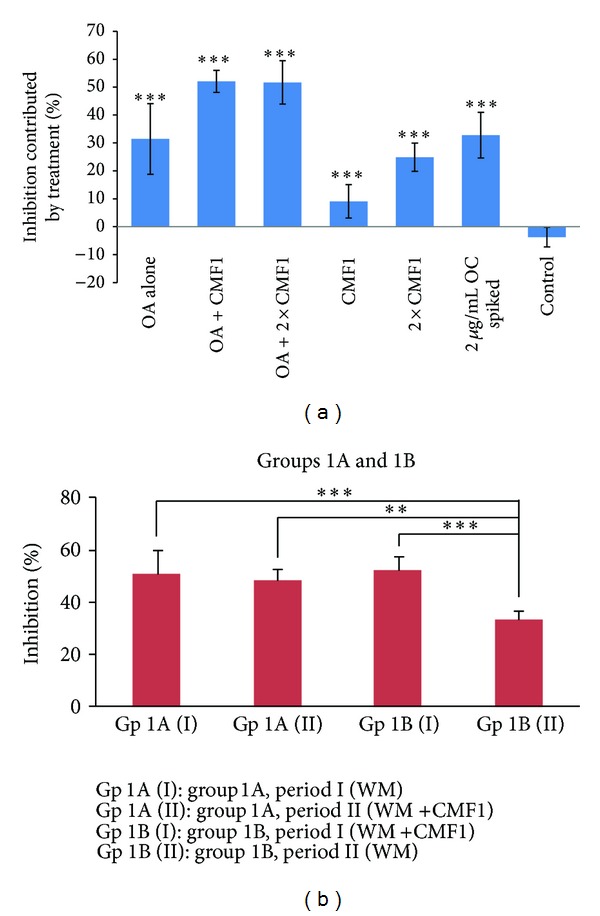
Comparison of inhibitory effect on virus replication of H3N2 of *ex vivo* plasma samples of (a) Rat treatment groups (*n* = 10 ~ 12 in each group) with all six treatment groups significantly different from control group (***P < 0.01), and (b) Human studies groups 1A and 1B collected at 2 h after drug administration on Day 5 (*P < 0.05; **P < 0.01; ***P < 0.001).

Inhibitory effects of ultrafiltrated plasma collected from the rats in all six Groups on the replication of human influenza A virus (H3N2) are shown in Figure 4. The inhibition effects from all treatment groups were significantly different from those of the control group (Group 6) (*P* < 0.01). The antiviral activities of Group 1 treated with OA alone were found to be significantly enhanced when OA was used in combination with CMF1 at the dose of 1.95 mg/kg (Group 2) (*P* = 0.006) or 3.90 mg (Group 3) (*P* = 0.008). Treatment with CMF1 alone (Groups 4 and 5) seemed to be able to exhibit significant antiviral activities in a dose-dependent manner. Inhibition effects from OA in combination with CMF1 (Groups 2 and 3) were comparable to those from the positive control with plasma spiked with OC.

#### 3.2.2. Findings from Human Studies

Figure 4 also shows the comparison of the inhibitory effects on virus replication of H3N2 strain of human plasma samples collected at 2 h after drug administration on day 5 from both groups and at different treatment sessions. By comparing the data of Group 1A, no significant difference in the inhibitory effect was observed between WM and WM + CMF1 treatments. On the other hand, subjects treated with WM in the second period, that is, Group 1B (II), had significantly lower (*P* < 0.001) inhibitory effect than those with WM + CMF1 treatment. In fact, the antiviral activity in this Group 1B (II) was also significantly lower than Group 1A with either WM (*P* < 0.001) or WM + CMF1 (*P* < 0.01) treatment. Similar trend was also observed in 0 h data.

## 4. Discussions

In order to mimic the clinical practice of both OA and CMF1, their human equivalent doses (OA at 30 mg/kg, CMF1 at 1.95 and 3.90 g/kg), dosing frequency (5-day dosing regimen), and dosing methods (oral) have been adopted for the current rat study. In addition, OA and CMF1 have been given in 2 h apart in order to mimic the clinical practice recommended for combination administrations of western and Chinese medicines in Hong Kong.

Our animal pharmacokinetic study indicated that CMF1 can significantly decrease OC concentration and urinary excretion, possibly resulting from a decrease of absorption or inhibition of presystemic metabolism of OA. Based on our *in vitro* and *in situ* intestinal perfusion studies, CMF1 was found to inhibit carboxylesterase activity both in the plasma and liver without a change of OA accumulation in the mesenteric vein. Thus, the observed decrease in OC concentration *in vivo* from administration of CMF1 is most likely a result of inhibition of pre-systemic OA metabolism by CMF1 at the site of mesenteric-portal vein area rather than a decrease of its absorption at the gastrointestinal site. Further studies on the specific components from CMF1 that play the major role in the inhibition of OA hydrolysis are warrant explaining such phenomenon. Although the *in vitro* incubation of CMF1 with OA in plasma may not entirely reflect the *in vivo* situation since not every CMF1 component could be absorbed as it appears in the extract, such study is used to preliminarily investigate the potential inhibition of hydrolysis of OA by CMF1, whereas the *in situ* intestinal perfusion study could reflect more of the absorption process in animal.

In rat studies, by comparson with those from OA alone, the *C*_max_, urinary recovery and AUC of OC, and the OC/OA AUC ratio in OA + CMF1 groups were significantly decreased (26%-27%) in a dose-dependent manner. The results presented in human study are also in line with the general decreasing trend when OA is co-administered with CMF1, but the extent of reduction is relatively small when compared to the animal studies. To study the effect of CMF1 on the pharmacokinetic parameters of OA and OC in human, a drug interaction approach with 90% CI is adopted in this study [12]. This approach is generally applicable to the interacting drug with one or a few active ingredients. For Chinese medicines formulation that constitutes at least tens of active compounds, it would not be feasible to single out each of them for the study. In this study, the CMF1 is considered to be the interacting drug. The co-administration of CMF1 did not alter the *C*_max_ and AUC_0–12 h_ of the active metabolite (OC) to a great extent. The point estimates were found to be near unity for both *C*_max_ and AUC_0–12 h_ of OC, with 90% CI of which within the 80%–125% criteria. The *C*_max_ of OA was reduced to ~78% when OA was co-administered with CMF1. This interaction was deemed unlikely to be clinically relevant, as OA is the inactive form (i.e., the prodrug). More importantly, the AUC_0–12 h_ of OC (the active metabolite) and the OC/OA AUC_0–12 h_ ratio (metabolic ratio) are similar between WM and WM + CMF1. The co-administration of CMF1 tends to lower (~14%) 12 h cumulative amount of OC excreted in urine but did not significantly change their pharmacokinetics parameters in plasma. However, care should be considered for renal impaired patients.

To compare the antivirus effects of OA alone or in combination with CMF1 present in rat plasma by plaque reduction assay, plasma sample was required to be ultracentrifuged followed by dilution with maintenance medium in 1 : 500 to avoid cytotoxicity to MDCK cells by removing the protein or the other matrix from plasma. Numerous plasma sample preparations methods have been tried in addition to ultracentrifugation such as liquid-liquid extractions with organic solvents or solid phase extractions of plasma samples followed by evaporating with nitrogen and reconstitute with buffer. Ultracentrifugation of collected plasma samples turns out to be the most efficient method to provide the least cytotoxicity to the MDCK cells with a single step of sample treatment. The enhancement of viral inhibition found in rats treated with OA + CMF1 (when compared with OA alone) was not observed in human study, probably due to the lower dosage of CMF1 used in human study. In addition, results on plaque reduction assay showed that there is a period effect on the inhibition of virus replication in Group 1B. It is noted that the 2 h post-dose plasma concentrations of OC between both treatments were similar (410–436 ng/mL). As shown in Table 3, the mean *C*_trough_ of OC was around 200 ng/mL (equivalent to ~704 nM) for both WM and WM + CMF1 treatments, which is over 1000-fold higher than the inhibitory concentrations (IC_50_) of OC against H3N2 (0.2–0.6 nM) or over 3- to 20000-fold higher than those against influenza virus strains (0.01–69.2 nM) [14]. It is expected that the concentration of OC (the active metabolite) would not be significantly reduced with the co-administration of WM + CMF1 and even a decreased inhibitory effect is observed.

Although the current study also indicated that CMF1 appeared to inhibit OA absorption and metabolism, combination of CMF1 with OA led to enhanced viral inhibition of OA as demonstrated by both rat and human studies. Mechanistic study in rat *in situ* intestinal perfusion demonstrated that CMF1 exhibited similar effects as our *in vivo* pharmacokinetic findings with inhibition on the formation of OC and no effect on the blood concentration of OA, which is also consistent with our *in vitro* rat plasma inhibition results. This further confirms the necessity to simultaneously monitor the western drug's pharmacokinetics and overall pharmacodynamics changes for such herb-drug interaction studies since their changes could be contradictory to each other. Such discrepancy is mainly due to the potential contribution of the pharmacodynamics activities from the multicomponents containing Chinese medicines, whose *in vitro* and *in vivo* levels could barely be monitored.

In addition, our present study showed for the first time an enhanced *in vivo* antiviral effect (using plaque reduction assay for* ex vivo* plasma samples) against influenza A virus (H3N2) when a CM formula, CMF1, was combined with OA. Thus enhanced effect from OA + CMF1 was observed despite a decrease in OC plasma concentration. CMF1 itself was also found to possess antiviral effect in a dose-dependent manner. The significant enhancement of antiviral effect by addition of CMF1 to OA may provide a new therapeutic approach for the treatment of resistant avian influenza in the future. Further study on the mechanism of its antiviral effect of CMF1 would warrant the translation of our current findings to the clinical practice.

## 5. Conclusion

The results show that co-administration with CMF1 in rat and Chinese male healthy volunteers had no clinically significant effect on the pharmacokinetics of OA and OC, although a generally lower trend was observed in both rat and human studies. Both OA and CMF1 were found to be well tolerated. Thus, the combination therapy of WM (75 mg bid for 5 days) and CMF1 (10 g extract per dose, bid for 5 days) in human at the recommended dosages is therefore considered to be safe and without significant pharmacokinetic consequences. The co-administration of OA and CMF1 can be complementary to each other for the treatment and prophylaxis of influenza.

## Figures and Tables

**Table 1:** The herbal composition of the CMF1.

Latin name	Chinese (pinyin) name
*Flos Chrysanthemi *	Ju Hua
*Flos Lonicerae Japonicae *	Jin Yin Hua
*Folium Mori *	Sang Ye
*Fructus Arctii *	Niu Bang Zi
*Fructus Forsythiae *	Lian Qiao
*Herba Lophatheri *	Dan Zhu Ye
*Herba Menthae *	Bo He
*Radix Et Rhizoma Glycyrrhizae *	Gan Cao
*Radix Platycodonis *	Jie Geng
*Rhizoma Phragmitis *	Lu Gen
*Semen Armeniacae Amarum *	Ku Xing Ren
*Semen Sojae Praeparatum *	Dan Dou Chi
*Spica Schizonepetae *	Jing Jie Sui

**Table 2:** Pharmacokinetic parameters of oseltamivir (OA) and oseltamivir carboxylate (OC) in rats after oral administration of OA alone (30 mg/kg) (Group 1) or OA (30 mg/kg) in combination with CMF1 at 1.95 mg/kg (Group 2) or at 3.90 mg/kg (Group 3), bid for 5 days.

Analyte	PK parameters	Group 1 (*n* = 10)	Group 2 (*n* = 11)	Group 3 (*n* = 10)
OA	*T*_max_ (min)	33.00 ± 19.75	35.45 ± 24.44	51.00 ± 14.49*
	*C*_max_ (*μ*g/mL)	1.16 ± 0.30	0.94 ± 0.13^#^	0.95 ± 0.23
	*t*_1/2, *λz*_ (min)	103.18 ± 8.77	100.91 ± 17.72	99.17 ± 11.63
	AUC_0–8 h_ (*μ*g∗min/mL)	199.85 ± 56.88	172.16 ± 23.70	195.56 ± 35.38
	AUC_0–inf_ (*μ*g∗min/mL)	209.14 ± 60.23	180.00 ± 26.40	213.28 ± 38.76
	*V*_*d*, *λz*_/F (L/k)g	23.39 ± 8.17	24.44 ± 3.75	20.75 ± 4.46
	CL/F (mL/min/kg)	158.34 ± 59.61	169.92 ± 24.63	144.75 ± 25.39
	CL renal (mL/min)	6.10 ± 1.32	5.47 ± 1.21	5.43 ± 0.97
	Renal recovery (% of dose)	15.84 ± 2.88	13.39 ± 3.07^#^	14.02 ± 1.41

OC	*T*_max_ (min)	105.00 ± 32.40	106.36 ± 20.63	120.00 ± 24.49
	*C*_max_ (*μ*g/mL)	1.65 ± 0.40	1.36 ± 0.28^#^	1.19 ± 0.23**
	*t*_1/2, *λz*_ (min)	151.70 ± 25.10	146.45 ± 19.10	156.83 ± 16.78
	AUC_0–8 h_ (*μ*g∗min/mL)	418.81 ± 90.86	344.13 ± 91.46^#^	304.84 ± 40.32**
	AUC_0–inf_ (*μ*g∗min/mL)	485.45 ± 113.03	395.67 ± 109.41^#^	359.21 ± 41.30**
	CL renal (mL/min)	6.33 ± 1.27	6.27 ± 1.76	6.34 ± 0.69
	Renal recovery (% of dose)	35.26 ± 5.40	29.73 ± 7.33^#^	25.94 ± 3.09**

OC/OA	AUC_0–8 h_	2.28 ± 0.85	2.03 ± 0.60	1.60 ± 0.35*
	AUC_0–inf_	2.51 ± 0.86	2.22 ± 0.61	1.73 ± 0.35*

*P < 0.05 and **P < 0.01 in comparison with those in Group 1; ^#^0.05 < *P* < 0.08 in comparison with those in Group 1.

**Table 3:** Summary of pharmacokinetic parameters following the administration of (a) WM and (b) WM + CMF1 in Chinese male healthy volunteers.

Analytes	PK parameters^a,b^	Treatment	
		WM (*n* = 14)	WM + CMF1 (*n* = 14)
OA	*C*_trough_ (ng/mL)	1.86 ± 1.72	2.36 ± 0.83
	*C*_max_ (ng/mL)	67.9 ± 27.1	51.3 ± 17.4
	AUC_0–12 h_ (ng·h/mL)	134.4 ± 41.1	127.8 ± 28.8
	*T*_max_ (h)	0.61 ± 0.21	0.93 ± 0.51
	*T*_1/2_ (h)	1.98 ± 0.40	2.94 ± 0.74
	12 h Cum. amt. in urine (*μ*g)	2883 ± 985	2810 ± 743
	Renal clearance (L/h)	22.1 ± 6.8	22.4 ± 5.2

OC	*C*_trough_ (ng/mL)	206.9 ± 67.7	195.3 ± 51.2
	*C*_max_ (ng/mL)	535.6 ± 102.2	487.4 ± 82.7
	AUC_0–12 h_ (ng·h/mL)	4585 ± 1155	4142 ± 783
	*T*_max_ (h)	4.21 ± 0.97	3.50 ± 0.94
	*T*_1/2_ (h)	5.16 ± 1.27	5.70 ± 1.35
	12 h Cum. amt. in urine (*μ*g)	72851 ± 15312	62087 ± 10741
	Renal clearance (L/h)	16.3 ± 3.8	15.4 ± 3.2

OC/OA	AUC_0–12 h_ ratio	37.0 ± 14.4	34.2 ± 10.7
	12 h Cum. amt. ratio	27.2 ± 8.4	23.2 ± 5.9

^a^ *C*_trough_: plasma concentration of analyte at 12 h postdose; *C*_max_: plasma concentration of analyte corresponding to *T*_max_; *T*_max_: time of maximum observed concentration; AUC_0–12 h_: area under curve from 0 to 12 h; *T*_1/2_: Terminal half-life; Cum. amt.: cumulative amount.

^b^Data was presented as arithmetic mean ± SD.

**Table 4:** Summary of geometric mean, geometric mean ratio, and 90% confidence internal (90% CI) of pharmacokinetic parameters between WM and WM + CMF1.

Analyte	Parameters	Geometric mean		GM Ratio, %^b^	90% CI, %^c^
		WM (*n* = 14)	WM + CMF1 (*n* = 14)		
OA	*C*_max_ (ng/mL)	62.42	48.81	78.19	60.82–100.51
	AUC_0–12 h_ (ng·h/mL)	129.86	124.76	96.08	88.48–104.33
	12 h Cum. amt. (*μ*g)^a^	2745.53	2726.98	99.32	85.56–115.30

OC	*C*_max_ (ng/mL)	526.27	480.59	91.32	84.81–98.33
	AUC_0–12 h_ (ng·h/mL)	4453.91	4067.40	91.32	83.80–99.52
	12 h Cum. amt. (*μ*g)^a^	71215.61	61224.10	85.97	76.64–96.44

OC/OA	AUC_0–12 h_ ratio	34.30	32.60	95.05	85.64–105.50
	12 h Cum. amt. ratio	25.94	22.45	86.56	76.49–97.94

^a^12 h cumulative amount of analyte in urine.

^b^Geometric mean ratio of (WM + CMF1)/WM.

^c^90% CI criteria of 80%–125% [10].

## References

[1] He G, Massarella J, Ward P (1999). Clinical pharmacokinetics of the prodrug oseltamivir and its active metabolite Ro 64-0802. *Clinical Pharmacokinetics*.

[2] Oo C, Barrett J, Hill G (2001). Pharmacokinetics and dosage recommendations for an oseltamivir oral suspension for the treatment of influenza in children. *Paediatric Drugs*.

[3] Oo C, Hill G, Dorr A, Liu B, Boellner S, Ward P (2003). Pharmacokinetics of anti-influenza prodrug oseltamivir in children aged 1–5 years. *European Journal of Clinical Pharmacology*.

[4] Massarella JW, He GZ, Dorr A, Nieforth K, Ward P, Brown A (2000). The pharmacokinetics and tolerability of the oral neuraminidase inhibitor oseltamivir (Ro 64-0796/GS4104) in healthy adult and elderly volunteers. *Journal of Clinical Pharmacology*.

[5] Abe M, Smith J, Urae A, Barrett J, Kinoshita H, Rayner CR (2006). Pharmacokinetics of oseltamivir in young and very elderly subjects. *Annals of Pharmacotherapy*.

[6] Yang MS, Law FC, Wong RN, Mak NK, Wei XY (2012). Interaction between oseltamivir and herbal medicines used for treating avian influenza. *Hong Kong Medical Journal*.

[7] (2010). *Chinese Pharmacopoeia 2010*.

[8] Lindegardh N, Davies GR, Tran TH (2007). Importance of collection tube during clinical studies of oseltamivir. *Antimicrobial Agents and Chemotherapy*.

[9] Lindegardh N, Davies GR, Hien TT (2006). Rapid degradation of oseltamivir phosphate in clinical samples by plasma esterases. *Antimicrobial Agents and Chemotherapy*.

[10] Chang Q, Chow MSS, Zuo Z (2009). Studies on the influence of esterase inhibitor to the pharmacokinetic profiles of oseltamivir and oseltamivir carboxylate in rats using an improved LC/MS/MS method. *Biomedical Chromatography*.

[11] Smee DF, Huffman JH, Morrison AC, Barnard DL, Sidwell RW (2001). Cyclopentane neuraminidase inhibitors with potent in vitro anti-influenza virus activities. *Antimicrobial Agents and Chemotherapy*.

[12] Zhou L, Chow MSS, Zuo Z (2009). Effect of sodium caprate on the oral absorptions of danshensu and salvianolic acid B. *International Journal of Pharmaceutics*.

[13] US Food and Drug Administration Guidance for industry. Drug interaction studies—study design, data analysis, and implications for dosing and labeling.

[14] Davies BE (2010). Pharmacokinetics of oseltamivir: an oral antiviral for the treatment and prophylaxis of influenza in diverse populations. *Journal Antimicrobial Chemotherapy*.

